# Considering sampling bias in close‐kin mark–recapture abundance estimates of Atlantic salmon

**DOI:** 10.1002/ece3.7279

**Published:** 2021-04-06

**Authors:** Sebastian Wacker, Hans J. Skaug, Torbjørn Forseth, Øyvind Solem, Eva M. Ulvan, Peder Fiske, Sten Karlsson

**Affiliations:** ^1^ Norwegian Institute for Nature Research Trondheim Norway; ^2^ Department of Mathematics University of Bergen Bergen Norway

**Keywords:** abundance estimation, Atlantic salmon, census size, close‐kin mark–recapture, Lincoln‐Petersen, mating success, mature male parr, population size, reproductive success, sampling bias

## Abstract

Genetic methods for the estimation of population size can be powerful alternatives to conventional methods. Close‐kin mark–recapture (CKMR) is based on the principles of conventional mark–recapture, but instead of being physically marked, individuals are marked through their close kin. The aim of this study was to evaluate the potential of CKMR for the estimation of spawner abundance in Atlantic salmon and how age, sex, spatial, and temporal sampling bias may affect CKMR estimates. Spawner abundance in a wild population was estimated from genetic samples of adults returning in 2018 and of their potential offspring collected in 2019. Adult samples were obtained in two ways. First, adults were sampled and released alive in the breeding habitat during spawning surveys. Second, genetic samples were collected from out‐migrating smolts PIT‐tagged in 2017 and registered when returning as adults in 2018. CKMR estimates based on adult samples collected during spawning surveys were somewhat higher than conventional counts. Uncertainty was small (CV < 0.15), due to the detection of a high number of parent–offspring pairs. Sampling of adults was age‐ and size‐biased and correction for those biases resulted in moderate changes in the CKMR estimate. Juvenile dispersal was limited, but spatially balanced sampling of adults rendered CKMR estimates robust to spatially biased sampling of juveniles. CKMR estimates based on returning PIT‐tagged adults were approximately twice as high as estimates based on samples collected during spawning surveys. We suggest that estimates based on PIT‐tagged fish reflect the total abundance of adults entering the river, while estimates based on samples collected during spawning surveys reflect the abundance of adults present in the breeding habitat at the time of spawning. Our study showed that CKMR can be used to estimate spawner abundance in Atlantic salmon, with a moderate sampling effort, but a carefully designed sampling regime is required.

## Graphical abstract

The aim of this study was to evaluate the potential of close‐kin mark–recapture (CKMR) for the estimation of spawner abundance in Atlantic salmon and how age, sex, spatial, and temporal sampling bias may affect CKMR estimates. Our study showed that CKMR can be used to estimate spawner abundance in Atlantic salmon, with a moderate sampling effort, but a carefully designed sampling regime is required.

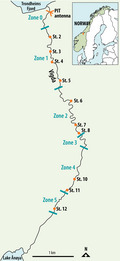

## 1 INTRODUCTION

Reliable knowledge of population size is the core of managing threatened species and essential for important questions in ecology and conservation. For example, population size affects the risk of extinction and the degree of inbreeding (Wright, 1931), but also interspecific processes such as predator–prey relationships (Begon et al., 2005). The estimation of population size remains challenging in many species, and in particular when populations are large and dispersed and when individuals are difficult to observe. With the increasing availability of neutral molecular markers (microsatellites and SNPs), genetic population size estimates have become feasible and are currently under rapid development. Abundance (*N_c_*) may be estimated with genetic analogues of mark–recapture studies in which individuals are sampled repeatedly and identified genetically (Lukacs & Burnham, 2005; Luikart et al., 2010). Recent advances have also been made to estimate abundance based on genetic kinship analysis and without repeated sampling of individuals (Bravington, Grewe, et al., 2016).

In close‐kin mark–recapture (CKMR), individuals are not physically marked. Instead, genotyped individuals “mark” their close‐kin, which may then be genetically identified (“recaptured”) in a second sample (Bravington, Skaug, et al., 2016; Skaug, 2017). This approach is straightforward when considering parent–offspring kinship for the estimation of adult abundance. Because each genotyped juvenile “marks” its two parents, adult abundance can be estimated from the number of parents detected in a sample of adults. In contrast to conventional mark–recapture, CKMR can be used when repeated sampling of adults is not possible or when all samples are collected from dead animals, for example in commercial fisheries (Bravington, Skaug, et al., 2016). Early applications of CKMR were limited to small populations, but decreasing genotyping costs and advances in parentage and sibship analysis allow for the method to be applied to large open populations, as recently demonstrated in southern bluefin tuna (*Thunnus maccoyii*) (Bravington, Grewe, et al., 2016) and white sharks (*Carcharodon carcharias*) (Hillary et al., 2018). A recent study validated CKMR for brook trout (*Salvenius fontinalis*) and found that abundance estimates were coherent with conventional counts and were associated with low uncertainty when a sufficient number of samples were collected (Ruzzante et al., 2019). CKMR has until now only been applied to a handful of species but is predicted to become an important tool in conservation and management (Bravington, Grewe, et al., 2016).

As many anadromous salmonids, Atlantic salmon (*Salmo salar*) are under severe pressure worldwide and reliable estimates of population size are crucial for their management. Anadromous Atlantic salmon migrate to the sea after a juvenile life‐stage in freshwater. After growth and maturation at sea, adults return to their natal river for spawning. This homing behavior leads to genetically distinct populations within and among rivers (Hendry et al., 2004). Ideally, the population forms the management unit for the species and for Atlantic salmon the most relevant estimate of population size is the number of adult individuals returning to a river (or part of a river) during the spawning season. This abundance metric is termed spawner abundance or escapement. A range of well‐established conventional (non‐genetic) methods exist for estimating spawner abundance, including direct counts carried out while snorkeling or wading along the river. Those methods are however dependent on certain conditions, such as good visibility and suitable water levels (Orell et al., 2011). Even under good conditions, not all fish are expected to be observable and parts of a river may not be covered. This is typically addressed by correcting the number of individuals observed by the estimated proportion of unobserved fish, but substantial uncertainty remains. Other approaches include the registration of Atlantic salmon entering a river with the help of cameras or sonars, often utilizing narrow sections (naturally or artificially created) in the rivers that allow cameras to detect all fish. Established conventional methods for the estimation of spawner abundance in Atlantic salmon are of high value for the monitoring and management of the species, but can be resource demanding, are not always feasible and may involve considerable uncertainty. CKMR may be a supplementary method and may in some cases be more suitable than established conventional methods.

Male salmon may mature precociously in freshwater, without migration into the sea. Those males are termed mature male parr and represent an alternative reproductive tactic. Whether males mature precociously is controlled by an interaction of genetic and environmental factors (Lepais et al., 2017). Mature male parr can be highly abundant and in sum fertilize a large proportion of eggs (up to 60%) (Herbinger et al., 2006; Martinez et al., 2000; Perrier et al., 2014). Mature male parr may thereby substantially contribute to the recruitment of the next generation and may have a substantial effect on the effective population size (Ferchaud et al., 2016; Johnstone et al., 2013; Saura et al., 2008). Nonetheless, those males are usually not included in conventional abundance estimates. A better understanding of the contribution of mature male parr to population size is important for management, but such estimates are difficult to obtain with conventional methods. CKMR is particularly suitable for including mature male parr in abundance estimates.

This study explores the potential of using CKMR to estimate the spawner abundance of Atlantic salmon, by for the first time applying it to a wild population. We estimate the abundance of adult Atlantic salmon in the River Vigda (Trøndelag, Norway) in the 2018 spawning season by nonlethal sampling of adults and their potential offspring emerging the following year. The genetic data comprise (a) 54 smolt PIT‐tagged in 2017 and returning as adults in 2018, (b) 67 adults sampled during surveys of spawners in 2018 and (c) 278 juveniles sampled with electrofishing in 2019 and being potential offspring from the 2018 spawning. Samples are genotyped at a high number of neutral molecular markers (SNPs), and the number of parent–offspring pairs is inferred from parentage analysis. The frequency of parent–offspring pairs is used to estimate adult abundance. The Atlantic salmon population in the River Vigda is well studied and therefore suited for the validation of CKMR. Each year, spawner abundance is estimated by conventional surveys, which also provide life‐history data that are highly valuable for the application and validation of CKMR. This study explores how the precision of CKMR estimates is affected by the sampling design and by sampling biases for spatial distribution, size, age, and sex of the sampled fish. This is an important step in the development and validation of CKMR for Atlantic salmon, but also in developing similar methodology for other fish species in both river and lake ecosystems.

## 2 METHODS

CKMR estimates of 2018 spawner abundance required genetic samples of adults returning to the river in autumn 2018 and of their potential offspring. Adult samples were obtained in two ways. First, scale samples were collected from adults caught during conventional spawning surveys (*Survey samples* hereafter). Second, genetic samples were collected during PIT‐tagging of smolt in 2017 and tagged individuals returning as adults in 2018 were registered with PIT antennas at the river entrance (*PIT samples* hereafter). Genetic samples of potential offspring were obtained by electrofishing of 0 + juveniles in 2019. CKMR estimates were compared with conventional counts of spawners.

### 2.1 Conventional spawning surveys

**FIGURE 1:**
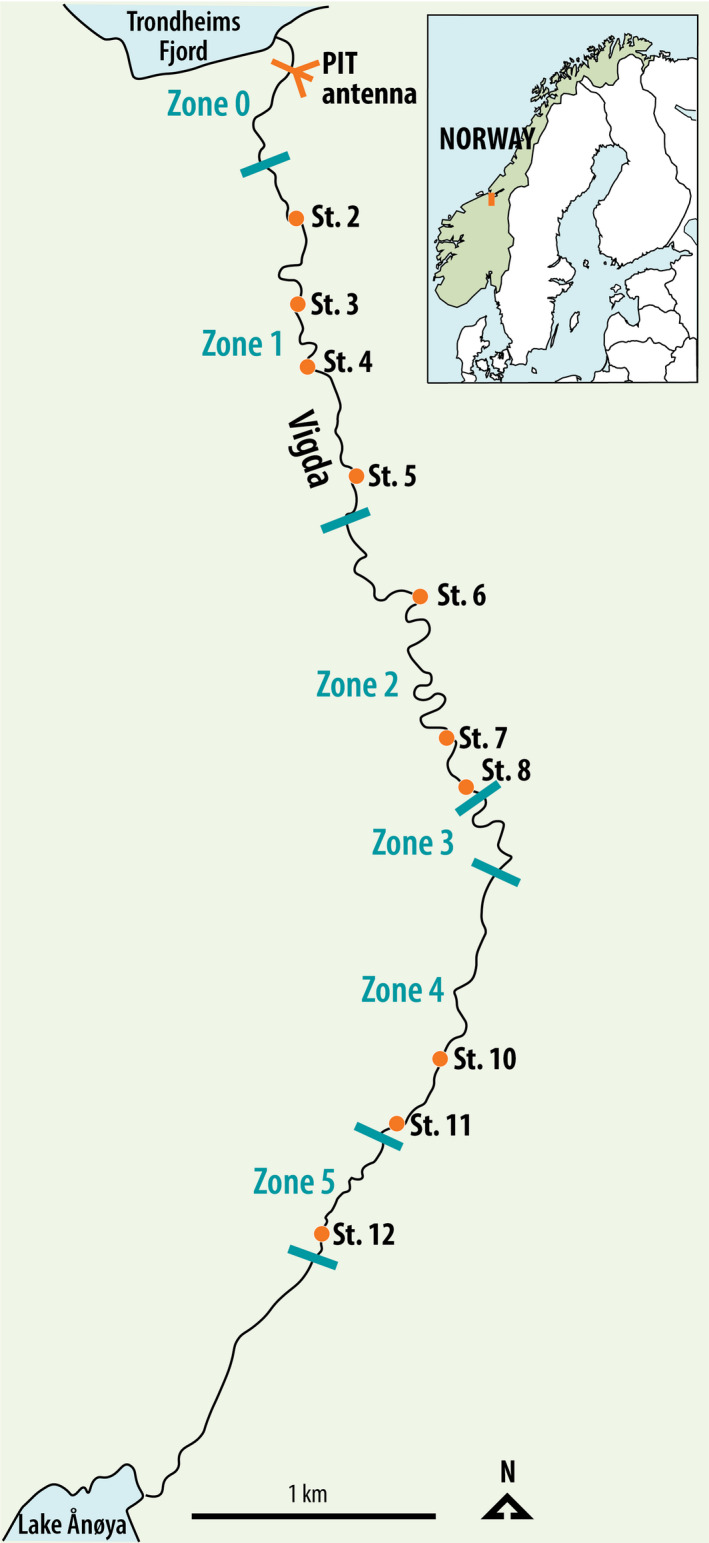
Sampling locations for adult (autumn 2018) and juvenile (summer 2019) salmon in River Vigda. Juveniles were sampled at ten electrofishing stations (St. 2–St. 12), and adults were sampled throughout the anadromous part of the river, divided into five zones (Zone 1–Zone 5). PIT‐tagged adults were registered by permanently installed antenna at the river entrance

The spawner abundance of adult Atlantic salmon in the River Vigda was estimated in conventional surveys in autumn 2018 (18–19 October). A team of three to five persons waded systematically through the riverbed at night‐time, using strong light to detect adult Atlantic salmon. Encountered fish were determined as either anadromous brown trout (*Salmo trutta*) or Atlantic salmon and based on their size, Atlantic salmon were categorized as small (<3 kg), medium (3–7 kg), and large (>7 kg). When possible, also the sex was determined. Spawning surveys started approximately 500 m upstream of the river mouth and proceeded upstream to the end of the anadromous section (Figure 1). The lowermost 500 m of the river (Zone 0) was too deep to wade and to count salmon. The survey covered a total of ca. 8,800 m of the river. Spawning surveys were limited by reduced visibility in 2018, and this was particularly the case in the lower part of the river (Zone 1 and Zone 2; Figure 1).

A total of 319 salmon were observed in the River Vigda during spawning surveys (Table 1). Corrections based on the estimated proportion of salmon successfully counted gave a total abundance of 402 salmon present in the river (CI: 375–432; Figure 2, 95% CI based on a simulation model developed to assess the size of the spawning population [Forseth et al., 2013]). The observations were corrected by expert judgment assuming that between 70% and 90% (triangular distribution with a modal value of 80%) of the fish on the surveyed river stretches were observed (in accordance with Forseth et al., 2013; Anon, 2009) and that 95% of the salmon in the river were on the surveyed stretches and 5% were in the lower reaches were no surveys were made. When counts are in addition corrected for the proportion of fish that entered the river after surveys had taken place (9% of the PIT‐tagged salmon entered the river after conventional spawning surveys took place), the total estimated number of salmon present during the spawning season was 439 (CI: 409–472; Figure 2).

**TABLE 1:** Numbers and proportions of salmon classified into three size categories (<3 kg, 3–7 kg, >7 kg) among a total of 319 salmon encountered during spawning surveys, among a subset of 67 salmon that were sampled and length measured during spawning surveys and among a subset of 252 salmon that were observed but not caught and for which size class was estimated from visual inspection

	*N*	<3 kg	3–7 kg	>7 kg
Total	319	212 (0.66)	101 (0.32)	6 (0.02)
Males	59	38 (0.64)	19 (0.32)	2 (0.03)
Females	76	31 (0.41)	43 (0.57)	2 (0.03)
Sampled	67	31 (0.46)	35 (0.52)	1 (0.01)
Males	24	18 (0.75)	6 (0.25)	0
Females	42	12 (0.29)	29 (0.69)	1 (0.02)
Observed‐only	252	181 (0.72)	66 (0.26)	5 (0.02)
Males	35	20 (0.56)	13 (0.38)	2 (0.06)
Females	34	19 (0.55)	14 (0.42)	1 (0.03)

Note

For each group of samples, proportions are also given for males and females separately for salmon that were sexed.

**FIGURE 2:**
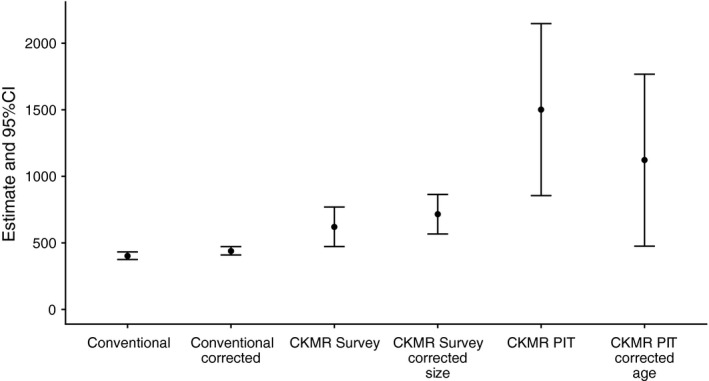
Abundance estimates for salmon in River Vigda in the 2018 breeding season. Conventional estimates are counts in spawning surveys, with corrected estimates taking into account that not all adults had entered the river at the time surveys were carried out. Close‐kin mark–recapture (CKMR) estimates are based on either scales sampled during spawning surveys (Survey) or on the registration of PIT‐tagged adults that entered the river (PIT). CKMR Survey estimates are corrected for size‐biased sampling (CKMR Survey corrected size). CKMR PIT estimates are corrected for sea‐age‐biased sampling, as only 1 SW adults were part of this sample

### 2.2 Survey samples

During conventional spawning surveys, 67 out of 319 encountered Atlantic salmon were caught with dip nets. Sampling was intended to be random with regard to sampling locality and demographic factors such as sex, age, and size but may have been affected by observability and catchability of individuals. Caught fish were sexed by visual inspection and length measured to the nearest cm, and weight class was inferred from an established length‐weight relationship (Figure S1A). For one individual, length was not recorded, but size class estimated from visual inspection. Scales were collected for genetic analysis and for reading of smolt and sea age. Scale readings revealed that most sampled males had spent one winter at sea, while most sampled females had spent two winters at sea (Table 2). All fish were handled carefully and released alive. Sampling of adult and juvenile fish was carried out with permission by the County Governor of Trøndelag.

**TABLE 2:** Proportions of male, female, and total salmon caught and sampled during spawning surveys that had spent one winter (1 SW), two winters (2 SW), three winters (3 SW), and four winters (4 SW) at sea

	*N*	1 SW	2 SW	3 SW	4 SW
Total	67	0.373	0.507	0.090	0.030
Males	24	0.750	0.250	0	0
Females	42	0.143	0.667	0.143	0.048

Sampling bias with regard to size and sex may be inferred from comparison of sampled fish with all fish encountered during spawning surveys. This comparison revealed a sampling bias for larger fish. About half of the sampled fish were of the larger size classes (≥3 kg) while that was the case for only about a third of all fish encountered (Table 1). Our data were not suitable for a reliable quantification of sampling bias for sex, because only a small part of the observed‐only (i.e., not sampled) fish were sexed and because larger fish were more frequently sexed than smaller fish (Table 1). Disregarding those limitations, a more female‐biased sex ratio among sampled salmon (63% females) than among all sexed salmon (56% females) suggests that sampling was moderately biased toward females (Table 1). Reliable sex‐ratio data were available for 1 SW (sea winter) PIT samples (see below), but it was unknown whether differences in sex ratio between PIT samples and 1 SW fish in survey samples were explained by sampling bias in survey samples. Alternatively, mortality or dispersal taking place between entry into the river and spawning surveys may have been sex‐biased. The sex ratio was female‐biased in PIT samples (63% females; Table 3), but male‐biased in 1 SW fish in survey samples (25% females; Table 3).

**TABLE 3:** Numbers of successfully genotyped salmon from River Vigda and results from genetic parental assignment of 278 juveniles

Sample type	Genotyped	Offspring assigned	Mean offspring	Parents detected	Mean offspring parents
Adults PIT	54	20	0.370 ± 0.12	10	2.000 ± 0.37
Males	20	11	0.550 ± 0.28	4	2.750 ± 0.63
Females	34	9	0.265 ± 0.11	6	1.500 ± 0.34
Adults survey	67	60	0.900 ± 0.16	29	2.069 ± 0.25
Males	24	24	1.000 ± 0.36	9	2.667 ± 0.65
Females	42	36	0.857 ± 0.17	20	1.800 ± 0.20
1 SW	25	15	0.600 ± 0.22	8	1.875 ± 0.40
2 SW	34	33	0.971 ± 0.26	15	2.200 ± 0.40
3 SW	6	2		1	
4 SW	2	1		1	
<3 kg	31	18	0.581 ± 0.18	10	1.800 ± 0.33
≥3 kg	36	42	1.167 ± 0.26	19	2.211 ± 0.35
Males < 3 kg	18	12	0.667 ± 0.28	6	2.000 ± 0.52
Males ≥ 3 kg	6	12	2.000 ± 1.13	3	4.000 ± 1.53
Females < 3 kg	12	6	0.500 ± 0.23	4	1.500 ± 0.29
Females ≥ 3 kg	30	30	1.000 ± 0.22	16	1.875 ± 0.25
Adults total	113	75	0.664 ± 0.11	37	2.027 ± 0.21

Note

Results are given for adult samples obtained from the registration of PIT‐tags at the river entrance (Adults PIT) and from scale samples collected from individuals caught during spawning surveys (Adults survey). For spawning surveys, results are also presented by sex, by sea age (1 SW, 2 SW, 3 SW, 4 SW) and by sex and size class (<3 kg, ≥3 kg). One individual caught during spawning surveys for which sex was not recorded is included in totals but not in the according sub‐categories. Means are reported ± one standard error. Note that sample sizes are small for some groups and for the smallest groups, means are not calculated.

### 2.3 PIT samples

Another sample of adult Atlantic salmon was obtained from a PIT‐tagging program in the River Vigda. Permanently installed antenna at the river entrance (Figure 1) registered any passage of tagged fish. Registration by two antennas allowed to infer the direction of movement, that is, whether individuals entered the river (upstream direction) or left the river (downstream direction). Individual PIT‐tagging needles were stored in lysis‐buffer after tagging each fish, and DNA was extracted from the small remains of tissue on the needles for genetic analysis. Genetic samples of PIT‐tagged adults entering the River Vigda in the 2018 spawning season were limited to fish that had spent one winter at sea (1 SW; smolt tagged 2017). Permission to PIT‐tag smolts was given by the Norwegian Food Safety Authority (FOTS ID 11313).

**FIGURE 3:**
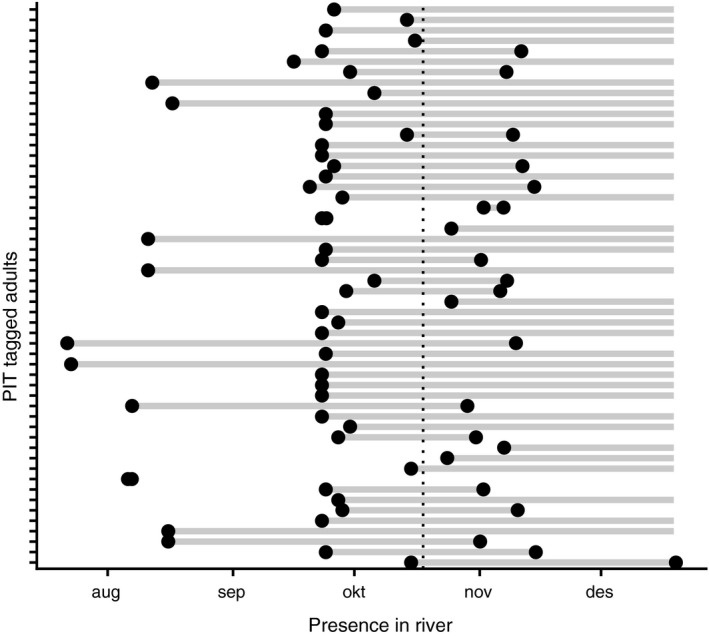
Presence of 54 PIT‐tagged 1 SW salmon in River Vigda in autumn 2018. Black dots indicate the date of registration at the river entrance in upstream and downstream direction, with gray lines indicating the duration of presence in the river. Duration is unknown for individuals for which no registration of leaving was made. The dotted vertical line represents when the spawning survey was conducted

A total of 54 salmon PIT‐tagged as smolt in 2017 were registered at the entrance of the River Vigda in autumn 2018 and successfully genotyped. Genetic sexing revealed that this sample consisted of 20 males and 34 females. Many of the PIT‐tagged fish made repeated passages across the antennae in upstream and downstream direction, potentially moving between freshwater and sea (data not shown). Figure 3 shows the presence of PIT registered salmon in the river over time, simplified to the longest continuous time period spent in the river. Stays in the river omitted in Figure 3 typically lasted few hours. Many of the PIT‐tagged fish were not registered as leaving the river after the spawning season, and it is unknown whether those individuals died in the river, lost their PIT‐tag during spawning (Dieterman & Hoxmeier, 2009) or whether their registration by antennae failed (Figure 3).

### 2.4 Juvenile samples

Juvenile samples were collected in autumn 2019 (10–11 October) by electrofishing (Solem et al., 2020). We collected 0+ juveniles, comprising offspring from the 2018 breeding year. The age of juveniles was determined based on body size (there is no or very low overlap in size between 0+ and older juveniles). Juveniles were collected at ten stations distributed throughout the entire anadromous part of the river. At each station, approximately 30 juveniles were collected (see Results). Juveniles were stored in ethanol, and tissue was later collected from gills for genetic analysis.

### 2.5 Genotyping

DNA was extracted from scale samples and PIT‐tagging needles using DNEASY tissue kit (QIAGEN). Samples were genotyped at a total of 192 SNPs using the EP1TM 96.96 Dynamic array IFCs platform (Fluidigm). The 192 SNPs included 15 mitochondrial and 177 nuclear SNPs, and out of those, 164 neutral loci were used for parentage analysis (Bourret et al., 2013). The sex of the PIT‐tagged smolt was determined genetically by amplifying a male specific sdY gene, according to Quemere et al. (2014). Because the sdY gene only amplifies in males, absence of amplification is interpreted as females, but can also be a result of a failed PCR amplification. Therefore, the sdY gene was amplified in a multiplex of markers for species identification developed by Karlsson et al. (2013) and absence of a sdY amplification was only interpreted as a female when all other markers in the PCR multiplex amplified well.

### 2.6 Parentage assignment

Parentage was assigned with the likelihood‐based approach implemented in the program COLONY (Jones & Wang, 2010). Assignment of parentage was run allowing for male and female polygamy and with an estimated probability of 0.2 that parents had been sampled, given the number of samples collected and the number of adult salmon present during conventional surveys. The proportion of mistyped loci was set to 0.001 (a conservatively high proportion). COLONY was run five times, with different seeds. Assignments to sampled adults were identical in those five runs, except for one juvenile. That juvenile was assigned in three out of five runs, and the assignment was kept for further analysis.

Two pairs of samples collected during spawning surveys had identical genotypes, indicating that two individuals were caught and sampled twice. One of the two samples from those two individuals respectively was excluded from further analysis. Fifty‐four of the adult salmon caught during conventional surveys were checked for PIT‐tags, and five were found to be PIT‐tagged. Genetic data verified that the genotypes obtained from PIT marking and scale samples respectively were identical. Identical genotypes were also found for additional three PIT‐tagged individuals that were caught during conventional surveys. Two of these three individuals were not checked for a PIT‐tag, while one individual was checked without detecting a PIT‐tag. For individuals that were both PIT‐tagged and sampled in spawning surveys, only one of the two samples (identical genotypes) was kept for parentage analysis, but the individuals were included in CKMR estimates for both survey samples and PIT samples.

### 2.7 CKMR

CKMR estimates for spawner abundance were calculated with an analogue of the Lincoln‐Petersen estimator, as described by Bravington, Grewe, et al. (2016):

(1) $${\hat{N}}_{\text{adult}}=\frac{2m_{J}m_{A}}{P},$$

where *m*_J_ and *m*_A_ are the number of juveniles and adults genotyped and *P* is the number of parent–offspring pairs detected. In this approach, each juvenile tags its two parents and the number of juveniles is therefore multiplied by two. The number of parent–offspring pairs represents the recaptures. Multiple juveniles may share parents, and the number of parent–offspring pairs may therefore exceed the number of adults genotyped. CKMR estimates may alternatively be calculated based on the number of unique parents identified in sibship analysis. That approach is presented in the Appendix S2 and abundance estimates and confidence intervals obtained from the two approaches are compared.

Confidence intervals for CKMR abundance estimates were calculated from the variance estimator for mark–recapture with sampling with replacement (Ricker, 1975; Whitmore, 2016):

(2) $$\hat{V}=\frac{{m_{A}}^{2}2m_{J}\left(2m_{J}‐P\right)}{P^{3}}.$$

We also estimated spawner abundance for males and females separately. While total spawner abundance may be affected by sex‐biased sampling of adults, male and female abundance is unaffected by sex‐biased sampling. Female spawner abundance was calculated as:

(3) $${\hat{N}}_{\text{female}}=\frac{m_{J}m_{\text{female}}}{P_{\text{female}}},$$

where *m*_female_ is the number of females genotyped and *P*_female_ is the number of dam‐offspring pairs detected. Because each juvenile tags a single dam, the number of juveniles is here not multiplied by two. Male spawner abundance was calculated analogously. The variance estimator for female and male spawner abundance was accordingly calculated as:

(4) $$\hat{V}=\frac{m_{\text{female}}^{2}m_{J}\left(m_{J}‐P_{\text{female}}\right)}{P_{\text{female}}^{3}}.$$

In this CKMR approach, variation in the number of offspring among adults does not affect the abundance estimate (Bravington, Grewe, et al., 2016; Bravington, Skaug, et al., 2016). An important requirement of the approach is that adult sampling must be independent of reproductive success and thus of the likelihood of detecting their offspring in the juvenile sample. When sampling is demographically biased (size and age related), this may be related to mating success and the number of offspring detected and thereby affect the abundance estimate.

We calculated CKMR abundance estimates for PIT samples and survey samples separately. Sampling differed in location (river entrance vs. breeding habitat) and time (river entry vs. time of breeding). Abundance estimates based on PIT samples reflect the total number of adults entering the river, while estimates based on survey samples reflect the number of adults alive and present in the breeding habitat at the time survey samples were carried out. A difference between the two estimates may for example result from mortality between river entry and the time of spawning.

### 2.8 Correction for size‐ and age‐biased sampling

Sampling of adults was biased with regard to size and age and did therefore violate the requirement of sampling adults independently from their reproductive success (Bravington, Skaug, et al., 2016; Ruzzante et al., 2019). There was a sampling bias for larger fish in survey samples and PIT samples consisted exclusively of 1SW fish. CKMR abundance estimates were adjusted for this sampling bias following the approach described by Ruzzante et al. (2019). That approach provides unbiased CKMR abundance estimates when fecundity and sampling probability depend on age (Ruzzante et al., 2019). The adjustment of the CKMR estimator requires knowledge of the relative reproductive success of the different age classes (Ruzzante et al., 2019).

We adapted equation (2) in Ruzzante et al. (2019) to the sampling regime in our study. Age classes in the original equation were replaced by size classes. In Ruzzante et al. (2019), adults were sampled over several years, including the year before and the two years after the breeding year for which adult abundance was estimated. In our study, all adults were sampled in the year for which adult abundance was estimated, which simplified the original equation. Male and female size‐specific reproductive success was assumed to be the same.

Fish were classified as small, medium, and large, but fish of the large size class were rare (only a single large fish was sampled during spawning surveys). We therefore pooled medium and large fish for this analysis. For adults registered by PIT‐tags, body size was unknown and therefore inferred from sea age. Among individuals sampled in spawning surveys, all 1SW fish were of the smallest size class and all adults registered by PIT‐tags were therefore assigned to the smallest size class.

Adult abundance was estimated by CKMR adjusted for size‐biased sampling as:

(5) $${\hat{N}}_{\text{adult}}=\frac{2m_{J}}{P}\times \left(\frac{R_{S}}{\bar{R}}\times n_{S}+\frac{R_{\text{ML}}}{\bar{R}}\times n_{\text{ML}}\right),$$

where *n*_S_ is the number of small fish in the adult sample and *n*_ML_ is the number of medium and large fish in the adult sample. *R*_S_ and *R*_ML_ are the average number of offspring for adults of the respective size classes and $\bar{R}$ is the average number of offspring in the population:

(6) $$\bar{R}=\frac{R_{S}*n_{S,\text{population}}+R_{\text{ML}}*n_{\text{ML},\text{population}}}{n_{S,\text{population}}+n_{\text{ML},\text{population}}},$$

where *n*_S,population_ and *n*_ML,population_ are the numbers of small (S) and medium and large fish (ML) fish in the population. Numbers of small and medium and large fish in the population (i.e., the size distribution of the population) were obtained in spawning surveys, during which the size of all encountered fish (319 individuals) was recorded.

### 2.9 Spatially biased sampling

Juvenile Atlantic salmon have a limited dispersal during the first year after hatching and are not expected to be randomly distributed in the river with regard to the place of hatching at the age of 0+ (Allendorf & Phelps, 1981; Bacles et al. , 2018). Parent–offspring pairs are therefore more likely to be found among adults and juveniles sampled in the same part of the river than among randomly chosen pairs of adults and juveniles. This spatial dependence may bias CKMR estimates (Conn et al. , 2020), but only when the proportion of breeders sampled varies among parts of the river. To test whether the spatial distribution of juveniles was non‐random with regard to the place of hatching, we inspected sampling localities of adults and juveniles forming parent–offspring pairs. To test whether variable proportions of breeders were sampled in different parts of the river and whether this affected proportions of juveniles assigned to parents, we inspected the proportion of juveniles assigned parents across the ten juvenile sampling locations.

### 2.10 Mature male parr

Mature male parr were not sampled in this study, but may in sum sire considerable proportions of offspring (Herbinger et al., 2006; Martinez et al., 2000; Perrier et al., 2014). The sampled juveniles included offspring of both anadromous males and mature male parr and our CKMR abundance estimate pertains to the combined anadromous male and mature male parr population. Unbiased estimation of total male abundance would however require that anadromous males and mature male parr had equal individual mating success. In contrast, mature male parr are expected to have a lower individual mating success than anadromous males (Martinez et al., 2000; Perrier et al., 2014). This would translate into a sampling bias toward males with high mating success and an overestimation of parent–offspring pairs when sampling anadromous males only, and an underestimation of total male abundance. Notably, abundance and reproductive contribution of mature male parr do not affect our CKMR estimates of female abundance.

The collected data did not allow to estimate the abundance of mature male parr, because mature male parr were not sampled. However, under the assumption that sampling of anadromous salmon was unbiased with regard to sex, equal numbers of parent–offspring pairs would be expected for males and females respectively in the absence of reproductive contribution by mature male parr. A lower number of parent–offspring pairs for anadromous males than for anadromous females would indicate that an according proportion of juveniles were fathered by mature male parr. The total reproductive contribution (proportion of offspring sired) of mature male parr was estimated as:

(7) $$r_{\text{MMP}}=\frac{\left(P_{\text{female}}‐P_{\text{male},A}\right)}{P_{\text{female}}},$$

where *P*_female_ is the number of female parent–offspring pairs and *P*_male,A_ is the number of anadromous male parent–offspring pairs. This formula follows by rearranging the equation

(8) $$P_{\text{male},A}=P_{\text{female}}\times \left(1‐r_{\text{MMP}}\right).$$

## 3 RESULTS

### 3.1 Genotyping and parentage analysis

A total of 278 out of 292 juvenile salmon (95%) and all adult samples were successfully genotyped at a minimum of 80% of the 164 loci and were included in further analysis. The average number of successfully genotyped loci among included samples was 162.

In total, 75 out of 278 juveniles were assigned to sampled parents and 37 out of 113 adults were detected as parents (Table 3). Out of 75 assignments, 68 assignments were made without mismatch, six assignments were made with mismatch at one locus and one assignment was made with mismatch at three loci. Among all pairwise comparisons between adults and juveniles, excluding parent–offspring pairs, the average number of mismatching loci was 12.1 (range: 1–29; *N* = 31,339).

### 3.2 CKMR estimates for PIT samples and survey samples

CKMR abundance estimates assuming unbiased sampling were more than twice as large when calculated for PIT samples (adults registered by PIT‐tags) than when calculated for survey samples (adults caught during spawning surveys) (Figure 2). For survey samples, 60 detected parent–offspring pairs (Table 3) translated into an abundance estimate of 621 adult salmon (95% CI: 472–769; CV = 0.12). For PIT samples, 20 parent–offspring pairs (Table 3) translated into an abundance estimate of 1501 adult salmon (95% CI: 855–2147; CV = 0.22). The lower number of parent–offspring pairs (despite similar numbers of adult samples) led to a much wider confidence interval of abundance estimates for PIT samples than for survey samples (Figure 2). The estimate based on survey samples was 41% higher than the conventional abundance estimate for anadromous individuals (439 salmon), while the estimate based on PIT samples was more than three times higher than conventional abundance estimates (Figure 2).

The difference in CKMR abundance estimates resulted from a twice as large proportion of adults assigned parentage for survey samples (43%) than for PIT samples (19%) (Table 3; *z*‐test: *χ* ^2^ = 7.3, *p* =.007). For adults assigned parentage, there was no difference in the number of offspring assigned to each parent among the two types of samples (Table 3).

### 3.3 Effect of age‐bias in PIT samples

Adults registered by PIT‐tags were exclusively 1 SW salmon (smolt tagged 2017). This generated an underestimation of parent–offspring pairs, because 1 SW fish had on average fewer offspring assigned than fish that had spent two and more winters at sea (Table 3). We used the relative reproductive success of small and larger (≥3 kg) adults (Table 3) to adjust the CKMR abundance estimate (Equation 5), resulting in a reduced abundance estimate of 1,122 adult salmon (95% CI: 776–1768; CV = 0.29) (Figure 2). The age‐bias in PIT samples resulted in 34% overestimation of abundance (uncorrected: 1501 individuals, corrected: 1,122 individuals).

The corrected abundance estimate was 57% higher than the abundance estimate based on survey samples after correction for size‐biased sampling (Figure 2), suggesting that the difference between CKMR estimates based on the two types of adult samples was not explained by biased sampling. This is also evident from direct comparison of PIT samples with survey samples of the same sea age. The mean number of offspring assigned to PIT samples (all 1 SW) was lower than for 1 SW fish in survey samples (Table 3). The difference in offspring assigned between PIT samples and individuals <3 kg in survey samples was more pronounced in females (90% higher) than in males (20% higher) (Table 3). Confidence intervals of CKMR abundance estimates based on the two types of adult samples did however overlap (Figure 2).

### 3.4 Effect of size‐bias in surveys samples

In sampling of adults during spawning surveys, there was a bias toward larger fish. Larger salmon (≥3 kg) made up about half of the sampled individuals, but only about a third of all individuals encountered during spawning surveys (Table 1). At the same time, larger salmon had a higher mean number of offspring assigned (Table 3). Together, this led to an overestimate of parent–offspring pairs and thus an underestimate of the CKMR abundance. We used the relative reproductive success of small and larger (≥3 kg) adults (Table 3) to adjust the CKMR abundance estimate (Equation 5), resulting in a reduced abundance estimate of 715 adult salmon (95% CI: 567–864; CV = 0.11). This estimate was 15% higher than the uncorrected CKMR estimate for survey samples and 63% higher than the conventional abundance estimate (439 salmon; Figure 2).

### 3.5 Effect of sex bias in survey samples

Our data did not allow to correct for a potential effect of sex‐biased sampling on CKMR estimates, because we did not have reliable estimates for the population sex ratio or the size distribution within sexes (see Section 2.2). Abundance estimates by CKMR are however unaffected by sex‐biased sampling when calculated for males and females separately. For males, 24 parent–offspring pairs translated into an abundance estimate of 278 males (95% CI: 172–384; CV = 0.20). For females, 36 parent–offspring pairs translated into an abundance estimate of 324 females (95% CI: 225–423; CV = 0.16). The sum of abundance estimates for males and females (602 salmon) was similar (approximately 3% lower) to the total CKMR abundance estimate (621 salmon), suggesting that a potential sex bias in survey samples did not strongly affect abundance estimates.

### 3.6 Mature male parr

The collected data were used to estimate the total reproductive contribution of anadromous males and mature male parr respectively. There was a pronounced sex bias among the anadromous salmon sampled during spawning surveys (Table 1). Under the assumption that this correctly reflected the sex ratio of anadromous spawners, the higher number of parent–offspring pairs for females than for males (Table 3) indicated that 33% of all offspring were fathered by mature male parr (Equation 7). Alternatively, the difference in parent–offspring pairs between males and females may indicate female‐biased sampling during spawning surveys.

Assuming that 33% of the juveniles were fathered by mature male parr, the number of juveniles marking sires can be corrected to estimate the number of anadromous males only. Out of 278 juveniles genotyped, 185 (67%) would have been sired by and thus marked anadromous males. With 24 anadromous males genotyped and 24 parent–offspring pairs detected (Table 3), this translated into a CKMR abundance estimate of 185 anadromous males and together with the estimate on female abundance (324 females), a total estimate of 509 anadromous salmon, which was 16% higher than the conventional abundance estimate (439 individuals).

### 3.7 Effect of spatially biased sampling

**FIGURE 4:**
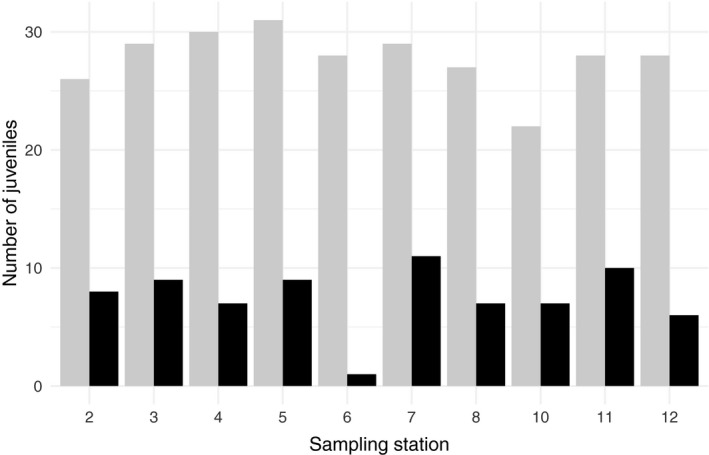
Number of juveniles collected at ten sampling stations (gray bars) in River Vigda and that were assigned to parents in genetic analysis (black bars)

Spatially biased sampling of adults may affect CKMR estimates when juvenile dispersal is limited. We found that juvenile dispersal was limited, as sampling localities of offspring were not independent from where parents were sampled (Table 4). Assigned offspring were in all cases located within the same zone (Figure 1) as their parents or downstream from their parents (Table 4). We tested for spatially biased sampling of adults by calculating the proportion of juveniles assigned to parents at each juvenile sampling station. The 278 genotyped juveniles were sampled at ten sampling stations spread throughout the river, with between 22 and 31 juveniles sampled at each station (Figure 4). Juveniles from all sampling stations were assigned to parents, with relatively equal proportions at all stations (0.21–0.38), except a low proportion at sampling station 6 (0.04) (Figure 4).

**TABLE 4:** Sampling location for adult salmon (and *N* of adults sampled) during a spawning survey in River Vigda relative to the sampling location of their offspring assigned in parentage analysis

	*N*	Assigned offspring				
		Zone 1	Zone 2	Zone 3	Zone 4	Zone 5
Adults Zone 1	12	12	0	–	0	0
Adults Zone 2	7	4	1	–	0	0
Adults Zone 3	15	3	12	–	0	0
Adults Zone 4	1	1	1	–	0	0
Adults Zone 5	32	6	2	–	14	4

Note

No juveniles were collected in Zone 3.

## 4 DISCUSSION

Our results show the potential and challenges in using CKMR for the estimation of spawner abundance of Atlantic salmon. With a moderate sampling effort, spawner abundance was estimated with reasonably high certainty. CKMR estimates were considerably higher than conventional estimates, but this may be explained by underestimation in conventional sampling, and that this method does not include mature male parr. Sampling of adults at the entry into the river (PIT samples) resulted in considerably higher abundance estimates than sampling in the breeding habitat (survey samples), and this may correctly reflect different adult abundances at the respective time and place. Sampling biases were evident in both types of adult samples, and their correction resulted in moderate changes in CKMR abundance estimates.

### 4.1 CKMR compared to conventional estimates

Abundance estimates from CKMR included mature male parr, which explains why the abundance estimate from CKMR was considerably higher than that from conventional spawning surveys. In addition, conditions for spawning surveys were relatively poor in 2018 and spawner abundance estimated was lower than in previous years, when estimates ranged from 500 individuals in 2016 (Solem et al., 2017) to more than 1,000 individuals in 2015 and 2017 (Solem et al., 2016, 2018). A relatively large proportion of individuals may thus have remained unobserved. The number of observed salmon was corrected for by the estimated proportion of unobserved individuals, but this correction was associated with a subjective uncertainty. It is therefore plausible that CKMR provided correct estimates of adult abundance. Complete counts do however not exist for the River Vigda and we cannot conclude with certainty how CKMR estimates and conventional estimates related to the true spawner abundance in the river.

When adult samples from spawning surveys were used, abundance was estimated with reasonably high certainty (CV < 0.15). The low level of uncertainty was achieved by a sampling regime that resulted in the detection of 60 parent–offspring pairs. The number of parent–offspring pairs determines to a large degree the uncertainty associated with CKMR abundance estimates (Bravington, Grewe, et al., 2016; Ruzzante et al., 2019). Uncertainty was accordingly larger when adult samples from PIT‐tagging were used, with only 20 parent–offspring pairs detected and a CV > 0.20. For samples from spawning surveys, the number of parent–offspring pairs and uncertainty in CKMR (CV) were similar to that obtained in previous applications in a tuna species (Bravington, Grewe, et al., 2016) and in brook trout (Ruzzante et al., 2019). A minimum of 50 parent–offspring pairs is considered a guidance in CKMR for obtaining a sufficiently low uncertainty (CV ≈ 0.15). Sparse sampling is realized in large populations, where 50 parent–offspring pairs would typically be found without any presence of half‐ or full‐siblings among the detected offspring (Bravington, Grewe, et al., 2016; Bravington, Skaug, et al., 2016). This was the case in the tuna study that detected 45 parent–offspring pairs among a total of 14,000 genotypes and that estimated census population size to approximately two million individuals (Bravington, Grewe, et al., 2016). In contrast, many of the 60 offspring detected in our study shared one and in some cases two parents with other offspring. This is expected in smaller populations, such as the studied salmon population or the brook trout populations studied by Ruzzante et al. (2019). In those cases, parent–offspring pairs cannot be treated as statistically independent and variance estimators should be adjusted accordingly (Bravington, Grewe, et al., 2016; Bravington, Skaug, et al., 2016), because half‐ and full‐siblings among offspring are expected to increase uncertainty. To our knowledge, variance estimates for CKMR in small populations have not yet been developed and this was beyond the scope of this study. We present an alternative CKMR approach in the Appendix S2, for which variance estimators are unaffected by sibship among juveniles. Results of that approach were very similar to the approach taken in the main part of the paper, suggesting that confidence intervals were correctly estimated despite the presence of half‐ and full‐siblings among offspring.

### 4.2 Sampling bias in survey and PIT samples

A central assumption of the CKMR approach applied in this study was that adult sampling was independent of reproductive success, that is, the expected number of parent–offspring pairs. Over‐proportional sampling of successful adults results in an overestimated number of parent–offspring pairs and an underestimated abundance. In accordance with previous studies (Dickerson et al. , 2005; Mobley et al. , 2019), we found differences in the mean number of offspring that were related to age (winters at sea), size, and sex. Any sampling bias for those demographic variables affects CKMR abundance estimates. In our study, it was possible to detect and estimate sampling biases for size and age because reliable data on true distributions of those demographic variables were available from conventional spawning surveys (Solem et al., 2019). It was therefore possible to correct abundance estimates for size‐ and age‐biased sampling, resulting in moderate corrections of the estimated abundance of salmon in the River Vigda. Separate estimation of male and female abundance suggested that sex‐biased sampling did not substantially affect abundance estimates. Those estimates were unaffected by a potential sex bias in sampling, and the sum of male and female abundances was very similar to our CKMR estimates for total abundance. There were however uncertainties associated to those data, and unbiased sampling would for example have allowed stronger inference on the contribution and abundance of mature male parr.

Spatial variation in sampling may bias CKMR estimates when dispersal is limited (Conn et al. , 2020). In Atlantic salmon, the spatial distribution of parents is expected to be related to the spatial distribution of their offspring, and in particular when juveniles are sampled at young age (Allendorf & Phelps, 1981; Bacles et al. , 2018). This was the case in our study, and we found that offspring were always found within the same area of the river or downstream of their parents. Given limited juvenile dispersal, biased sampling of adults may have resulted in biased abundance estimates (Conn et al. , 2020). We tested this by plotting the proportion of juveniles assigned to parents across sampling stations and found it to be relatively even, suggesting that adult sampling was balanced. While spatially biased sampling of juveniles would not bias the abundance estimate given uniform adult sampling, it would affect the confidence interval of the estimate. When juveniles are sampled in a more limited area, more juveniles are expected to share parents, which increases the variance estimator of the CKMR estimate (Appendix S2).

Our results showed the importance of a careful sampling regime in CKMR studies and how sampling biases may affect abundance estimates. In other systems or in the application of CKMR to Atlantic salmon in other rivers or with other types of data, more sophisticated methods may be appropriate to detect and correct for sampling biases. For example, our approach did not consider the statistical uncertainties in the estimation of the demographic composition of samples or in the estimation of reproductive success of the different classes of fishes. Our approach also made assumptions that would need further exploration. For example, differences in reproductive success between size classes in survey samples were used to correct for sea age‐biased sampling in PIT samples, under the assumption that the effect of sea age on reproductive success was the same as in survey samples. As a suitable alternative or complementary approach to corrections based on empirical data, especially when those are lacking or are limited, potential effects on sampling biases may be evaluated by simulations. This has recently been shown by Conn et al. (2020), with a focus on spatially biased sampling in CKMR studies.

### 4.3 Adult sampling method

We found very different results for PIT‐tagged adults than for adults sampled during spawning surveys. On average, only half as many offspring were assigned to PIT‐tagged adults than to adults sampled during spawning surveys and abundance estimates were accordingly higher when using PIT samples. There was large uncertainty for CKMR abundance estimates for PIT‐tagged adults, due to a low number of parent–offspring pairs, but adult sample size was similar for the two sampling methods. Differences were not explained by adult sampling bias, and we are not aware of studies suggesting that PIT tagging of smolt negatively affects adult freshwater survival or spawning success. Instead, differences in CKMR estimates may correctly reflect different abundances of salmon at the time and place of sampling. PIT‐tagged fish were a random sample of (one‐sea‐winter) salmon entering the river in the course of the entire breeding season. CKMR estimates based on PIT‐tagged salmon are therefore expected to estimate escapement, that is, the total number of adults returning to the river. Samples collected in spawning surveys were in contrast taken in the spawning habitat and in mid‐October, when spawning was ongoing. We have no indication that sampling during the spawning season negatively affected spawning success. Notably, any negative effect of sampling during spawning surveys would have reduced the difference in abundance estimates observed between the two adult sampling methods. CKMR estimates based on those samples are therefore expected to estimate the number of adults present in the breeding habitat during spawning and were also closer to the conventional estimates. A reduction from the first to the second abundance estimate may be explained by mortality taking place in the river and before spawning. The 2018 spawning season was exceptional in most Norwegian salmon rivers, with very little precipitation and with low water levels until late in the season. Very few salmon (28 reported individuals) were caught by angling in the River Vigda in 2018 (Solem et al., 2019), but the exceptional conditions in the river may have increased natural mortality. It seems however unlikely that (pre‐spawning) mortality alone explained the 40% decrease in abundance estimated from PIT samples to abundance estimated from spawning survey samples.

Alternatively, average spawning success may have differed between the two types of samples for other reasons than mortality. Individuals that were unsuccessful in reproductive competition may have been excluded from the mating habitat before spawning surveys took place. Those individuals may have gained no mating success or had on average lower mating success than the individuals encountered and sampled during spawning surveys. In contrast, any unsuccessful individuals would be represented in the sample of PIT‐tagged individuals. Differences in the number of offspring assigned between PIT samples and survey samples were much larger for females than for males, suggesting higher in river mortality or more pronounced exclusion from breeding in females than in males. This is also supported by the change in sex ratio from female‐biased in PIT samples to male‐biased in fish of the same sea age in survey samples.

### 4.4 Mature male parr

In our CKMR approach, mature male parr were “marked” by their offspring in the same way as anadromous males. Sampling of adults did in contrast not include mature male parr. As outlined in the results section, our sampling regime and the presumably lower mean individual reproductive success of mature male parr is therefore expected to lead to an underestimation of total male abundance (anadromous males and mature male parr) and of total population abundance. CKMR may be used to estimate the proportion of offspring fathered by mature male parr rather than anadromous males. In our study, we estimated this proportion to be 33%, but the estimate may have been strongly affected by sex‐biased sampling. If this proportion is estimated with greater certainty, it can be used to correct the CKMR abundance estimate for anadromous males and for the total abundance of anadromous adults. If in addition mature male parr were sampled, their abundance may be estimated with CKMR.

### 4.5 Potential of CKMR for Atlantic salmon management

Our study showed that CKMR can be used to estimate spawner abundance in Atlantic salmon. A moderate sampling regime involving approximately 350 genotypes was sufficient to estimate abundance in a population of about 700 spawners. CKMR has earlier also successfully been applied to coho salmon (Whitmore, 2016) and to chinook salmon (Rawding et al. , 2014). Those species differ in their reproductive biology from Atlantic salmon, and adults were sampled as carcasses after spawning, while juveniles were sampled by electrofishing. Together with the present study, those studies show that CKMR can be used to estimate spawner abundances in anadromous salmonids. CKMR also has a great potential for the estimation of abundance in other aquatic species, for which the currently available methods are even more limited. This includes many fish species in inland rivers and in lakes, as recently demonstrated for brook trout (Ruzzante et al. , 2019).

For smaller rivers like the River Vigda, sampling during spawning surveys was a good approach. Alternative sampling methods may include catch and release angling in autumn and bag net fisheries in the sea. A potentially large effect from this sampling is that it can affect mortality and reproductive success. It is therefore very important that the sampling is done carefully without harming the fish and it is probably also important to not sample the fish too close up to spawning as this might have a negative impact on their success. Tagged smolt only registered by antenna when they return appear in this respect as a very good method. Combining the two sampling approaches taken in this study may provide valuable information on the extent to which abundance is reduced from adults entering the river to adults taking part in spawning.

In conclusion, the close‐kin mark–recapture (CKMR) method is a useful tool for estimating the abundance of Atlantic salmon. It is important to avoid, or to correct for sampling bias and most importantly to design the sampling of potential parents in accordance with what they represent; escapement or number of spawners.

## CONFLICT OF INTEREST

None declared.

## AUTHOR CONTRIBUTION

**Sebastian Wacker:** Conceptualization (equal); Data curation (equal); Formal analysis (equal); Funding acquisition (equal); Investigation (equal); Methodology (equal); Project administration (equal); Writing‐original draft (lead). **Hans J. Skaug:** Conceptualization (equal); Methodology (equal); Writing‐review & editing (equal). **Torbjørn Forseth:** Conceptualization (equal); Funding acquisition (equal); Writing‐review & editing (equal). **Øyvind Solem:** Data curation (equal); Investigation (equal); Writing‐review & editing (supporting). **Eva M. Ulvan:** Data curation (equal); Investigation (equal); Writing‐review & editing (equal). **Peder Fiske:** Data curation (equal); Writing‐review & editing (equal). **Sten Karlsson:** Conceptualization (equal); Data curation (equal); Funding acquisition (equal); Investigation (equal); Methodology (equal); Writing‐review & editing (equal).

## Figures and Tables

**FIGURE A1:**
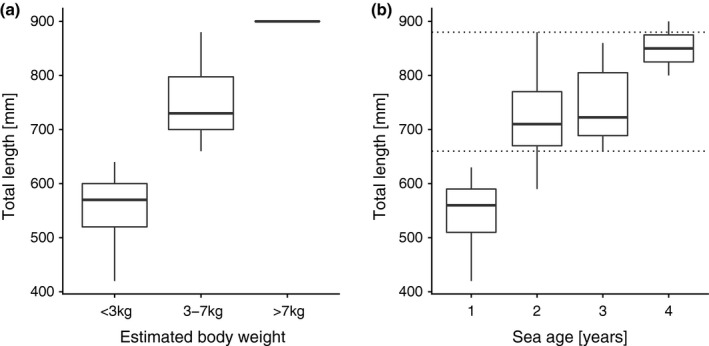
(A) Total length [mm] of salmon classified into three size categories (<3 kg, 3–7 kg, >7 kg) in 66 salmon caught during spawning surveys. (B) Variation in total length [mm] among salmon of different sea age in 66 salmon caught during spawning surveys. Dotted lines indicate the size limits (660 and 880 mm) for the estimation of body weight categories

**FIGURE A2:**
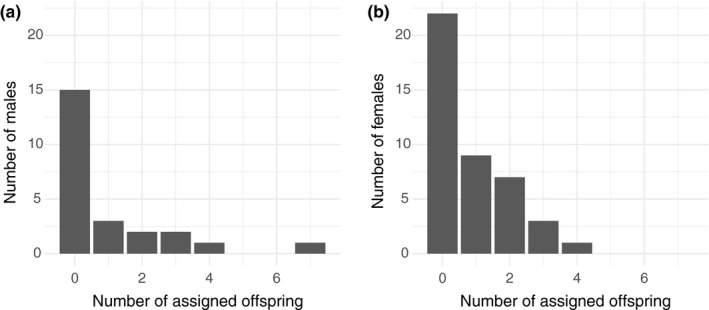
Distribution of the number of offspring assigned to males (A) and females (B) sampled during spawning surveys in River Vigda in autumn 2018

## Data Availability

Data on sampled adults and sampled juveniles including sampling location, sex, age, and size (where available), genotypes and results from parentage analysis have been archived at Dryad (https://doi.org/10.5061/dryad.12jm63xxb).

## APPENDIX 2

There are two approaches for estimating CKMR spawner abundance, termed “with replacement” and “without replacement” (Rawding et al., 2014, Whitmore, 2016). The “with replacement” approach refers to a situation where juveniles may share parents (half‐ and full‐siblings), and adults may therefore be marked and detected multiply. Drawing samples from the adults must therefore be done “with replacement,” to allow the detection of more than one mark per adult. The “with replacement” approach was applied and presented in the manuscript and here we present the “without replacement approach.”

In the “without replacement” approach, CKMR estimates are calculated from the number of unique parents marked by the juveniles. For example, four half‐siblings mark five parents (one parent of the first sex and four parents of the second sex), while four full‐siblings mark two parents. The required information on sibship and thereby total numbers of unique parents marked was obtained from the COLONY sibship analysis described in the main part of the paper. The 278 juveniles genotyped in our study were assigned to a total of 262 unique parents (including sampled adults and not sampled parents inferred by sibship analysis). CKMR estimates for spawner abundance were calculated with an analogue of the Lincoln–Petersen estimator, as described by Rawding et al. (2014):

$${\hat{N}}_{\text{adult}}=\frac{m_{J}^{\prime }m_{A}}{P^{\prime }},$$

where *m′_J_* is the number of unique parents detected in sibship analysis (for the juveniles genotyped), *m_A_* is the number of adults genotyped and *P′* is the number of sampled unique adults assigned parentage.

Confidence intervals for CKMR abundance estimates were calculated from the variance estimator for mark–recapture with sampling without replacement (Ricker, 1975, Whitmore, 2016):

$$\hat{V}=\frac{m_{A}m_{J}^{\prime }\left(m_{J}^{\prime }-P^{\prime }\right)\left(m_{A}-P^{\prime }\right)}{P^{\prime 3}}$$

**TABLE A1:** Table comparing CKMR abundance estimates, 95% confidence intervals (CI) and coefficients of variation (CV) calculated with two alternative approaches. Results from the “with replacement” approach are presented in the main part of the paper.

	Without replacement			With replacement		
	Abundance	CI	CV	Abundance	CI	CV
Survey samples	605	457–754	0.12	621	472–769	0.12
PIT samples	1,414	763–2,066	0.23	1,501	855–2,147	0.22
